# Surgical Treatment in Ulcerative Colitis, Still Topical: A Narrative Review

**DOI:** 10.7759/cureus.41962

**Published:** 2023-07-16

**Authors:** Eduard Slonovschi, Pratyusha Kodela, Monalisa Okeke, Sandeep Guntuku, Shanmukh Sai Pavan Lingamsetty

**Affiliations:** 1 Department of Surgery, Universitatea de Medicină și Farmacie Iuliu Haţieganu Facultatea de Medicină, Cluj-Napoca, ROU; 2 Department of Research, Shri B. M. Patil Medical College, Hospital and Research Center, Bijapur Liberal District Educational Association (BLDEA) University, Vijayapura, IND; 3 Department of Research, Drexel University College of Medicine, Philadelphia, USA; 4 Department of Internal Medicine, Mamata Medical College, Khammam, IND

**Keywords:** colorectal cancer (crc), surgical treatment, postoperative complications, • open surgery, preventive colectomy, total proctocolectomy, timing, ulcerative colitis (uc), inflammatory bowel disease, laparascopic surgery

## Abstract

In this paper, different studies were integrated to conclude the impact of ulcerative colitis (UC) on the patient's vital prognosis, specifically highlighting the association with colorectal cancer (CRC). These severe complications have led us to consider studying the role of preventive surgery in managing UC. This study reviewed total preventive colectomy in UC patients for preventing the onset of CRC, the role of surgery in UC management, and its potential as a definitive treatment for the condition. The study also emphasized the effectiveness of annual colonoscopic monitoring and preventive colectomy in reducing the incidence of colorectal cancer (CRC). It discussed the role of laparoscopic surgery in minimizing postoperative complications and highlighted that partial surgical resection of the colon can be a viable option, offering improved bowel function without increasing the risk of CRC-related mortality. Elective surgery has an important place in UC management by preventing the development of forms requiring emergency surgery. Although surgery can cure UC, it can lead to significant postoperative complications and adverse effects.

## Introduction and background

Ulcerative colitis (UC) is an idiopathic chronic inflammatory disorder of the colon characterized by the inflammation of the mucosa of the rectum that spreads proximally and continuously through part or all of the colon [1]. Relapsing and remitting symptoms are characteristic of UC [2]. It was K. Rokitansky who described UC for the first time in meticulous detail in 1842 [3]. S. Wilks and W. Mason described the pathology of the colon in UC for the first time in 1875 [3]. In UC, men and women are affected similarly, and the diagnosis is made in most cases at the end of adolescence or early adulthood [4,5]. However, the occurrence of UC symptoms and its subsequent diagnosis can be made at any age [5]. In recent decades, there has been a notable increase in the incidence of UC worldwide, particularly in developing countries. However, it is important to note that UC predominantly affects patients of Northern and Western European ancestry [6,7]. However, compared to the former data we had on inflammatory bowel disease, lately, there has been an increase in the incidence of inflammatory bowel disease in non-white populations [8]. A genetic component of inflammatory bowel disease triggers an immune reaction against gut microbes that causes inflammation in the gut wall. However, the exact cause of this disease remains unknown, even if genetic, environmental, and microbial factors might be involved [9-12]. UC can lead to complications like perforation, life-threatening hemorrhages, toxic megacolon, pseudopolyps, strictures, and CRC [13,14]. Without an alternative etiology, UC is diagnosed based on clinical presentation, endoscopic evaluation, and histological parameters [15]. The gold standard for diagnosing UC remains colonoscopy, although laboratory and radiographic findings can help [15]. However, the rise in UC incidence is paralleled by an increase in life expectancy and a reduction in patient mortality rates. This can be attributed to the therapeutic advancements made in the management of UC over the last few decades [5]. It is important to consider the role of total preventive colectomy in patients diagnosed with UC because once UC is diagnosed, the person's risk of CRC increases eight or 10 years later [16-18]. It is estimated that people with longstanding UC have a six to 10 times greater risk of developing CRC (i.e., a 10%-15% greater risk) compared to a population without UC [19]. Nevertheless, today, with increasingly efficient endoscopic techniques, regular colonoscopic monitoring is considered the safest way to treat UC patients at high risk of developing CRC [19]. Also, the cost of the different procedures is an important criterion. Nguyen et al., in their analysis, demonstrated that immediate colectomy is more effective and associated with lower costs compared to enhanced surveillance ($75,900 vs. $83,900) [20]. This study aims to emphasize the role of surgery in the management of UC, particularly considering the increase in UC incidence observed in recent decades. It emphasizes the importance of total preventive colectomy as a means to prevent the occurrence of CRC in patients with UC. Additionally, the study outlines the use of surgery as a definitive treatment option for UC.

## Review

CRC in UC: Pathologic development, progression, and course

The incidence and prevalence of UC vary geographically, and patients with UC are also more likely to develop CRC [21].

**Figure 1:**
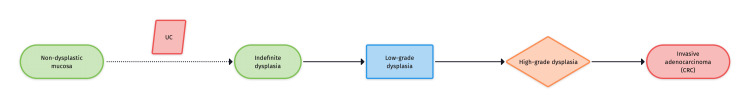
Pathogenesis of CRC production in UC. Figure credit: All authors. UC, ulcerative colitis; CRC, colorectal cancer

In UC, CRC begins with nondysplastic mucosa that progresses to indefinite dysplasia, low-grade dysplasia, and high-grade dysplasia, which leads to invasive adenocarcinoma (Figure 1) [22]. A combination of factors produced by the host immune response and the gut microbiome, gut microbiome products contribute to inflammatory and carcinogenic processes [23]. Thus, genetic (such as mutations) and epigenetic (such as methylation) modifications will be produced, which will lead to clonal expansions of somatic epithelial cells under the influence of surrounding stromal and immune cells [23]. DNA damage caused by chronic inflammation activates tumor-promoting genes and inactivates tumor suppressor genes due to oxidative stress [24]. As they progress, there is a progressive increase in oxidative damage markers and DNA double-strand breaks in the inflammation-dysplasia-carcinoma sequence [25]. Inflammation-induced carcinogenesis is often considered the prototype of CRC resulting from inflammatory bowel disease (IBD) [23].

In 2020, Olén et al. published a population-based cohort study carried out over the past 50 years on a sample of 96,447 patients with UC from Sweden and Denmark who were followed up for CRC incidence and CRC mortality and compared with matched reference individuals from the general population of Sweden and Denmark (*n *= 949,207) [26]. It was found that 1,336 cases of CRC were diagnosed in the UC cohort (1.29 per 1,000 person-years) and 9,544 cases of CRC in reference individuals (0.82 per 1,000 person-years) [26]. Also, 639 patients died of CRC in the UC cohort (0.55 per 1,000 person-years) compared to 4,451 patients who died of CRC in reference individuals (0.38 per 1,000 person-years) during the same period [26]. A person with UC is more likely to develop CRC and die from it than someone without UC [26]. The results of the above study can be compared with another study (a retrospective cohort) conducted by Venkataraman et al. in India in 2005, where over the last 25 years, the archived files of all UC patients who underwent colonoscopy and segmental biopsies were analyzed [27]. Five hundred thirty-two patients describing it were placed in the retrospective cohort [27]. The risk of developing CRC during the first 10 years of illness was zero [27]. The threat has increased for those with UC lasting between 10 and 20 years and corresponded to 2.3% for all patients with colitis and 4.4% for patients with pancolitis [27]. The risk has increased even more in patients with UC, lasting more than 20 years, with a chance of 5.8% and 10.2%, respectively [27]. Authors have concluded that the only risk factors significantly associated with developing CRC in patients with UC are the extent of colitis and a disease duration exceeding 10 years [27]. The study also concluded that patients with UC from India have a lower risk of developing CRC than those from the West (developed countries) [27]. The previous results are confirmed by the study of Zhang et al., who conducted a retrospective, monocentric study in 2015 over 12 years in China in a sample population of 642 cases with UC, followed until the appearance of their CRC confirmation [28]. It was found that CRC was diagnosed in UC at a percentage of 0.64 [28]. As in the previous study, they found that longer disease duration and extensive colitis are risk factors for developing CRC[28]. The study also concluded that the prevalence of CRC in China is lower than in the West (developed countries) [28]. Table 1 summarizes the studies related to UC complications.

**Table 1:** Summary of studies related to UC complications. UC, ulcerative colitis; CRC, colorectal cancer

References	Year of publication	Design	Number of participants	Population	Conclusion
Olén et al. [26]	2020	Population-based cohort study	96,447	Patients with UC from Sweden and Denmark	A person with UC is more likely to develop CRC and die from it compared to someone without UC.
Murphy et al. [34]	2016	Retrospective review	175	Patients undergoing surgery for UC in the United States	The incidence of CRC is low in patients with dysplasia if they postpone surgery for up to five years.
Zhang et al. [28]	2015	Retrospective, monocentric study	642	Patients with UC from China	Longer disease duration and extensive colitis are risk factors for CRC development. And the prevalence of CRC in China is lower than in the West.
Venkataraman et al. [27]	2005	Retrospective cohort	532	Patients with UC from India	The only risk factors significantly associated with developing CRC in patients with UC are the extent of colitis and the duration of the disease more of than 10 years. And patients with UC from India have a lower risk of developing CRC than patients from the West.
Lim et al. [35]	2003	Retrospective cohort	160	Patients with longstanding extensive UC	The diagnosis of low-grade dysplasia does not warrant prophylactic colectomy. Low-grade-dysplasia cases should not be ruled out for conservative management.
Ullman et al. [33]	2003	Retrospective cohort	46	Patients with UC diagnosed with low-grade flat dysplasia	Low-grade flat dysplasia in UC patients is a strong predictor of progression from dysplasia to advanced neoplasia. And we should recommend early colectomy.

CRC screening encompasses various methods that can be classified into two categories: invasive and non-invasive tests [29]. Non-invasive tests for CRC screening include stool-based tests, blood-based tests, and X-ray tests [29]. There are several stool-based tests available, including guaiac-based fecal occult blood testing (gFOBT), fecal immunochemical testing (FIT), and new fecal DNA testing (multi-target stool DNA) [29]. These tests can detect blood or cellular debris produced by vascularized polyps, adenomas, or cancers [30]. Radiological tests are represented by double-contrast barium enema, capsule endoscopy, and computed tomography colonography (CTC) [29]. They are responsible for visualizing and identifying advanced colonic polyps and cancers on radiographs and detecting extracolonic findings (by CTC) [29]. Colonoscopies and flexible sigmoidoscopies are invasive tests that offer direct visualization and detection of polyps and neoplasms and get pathology samples [29]. However, in the United States, colonoscopy has been introduced as the primary screening tool [29].

The role of preventive colectomy in UC

By analyzing previous studies, we have seen that the risk of developing CRC in patients with UC increases sharply with disease duration (>10 years) and extensive colitis [27]. In patients with longstanding and extensive colitis, prophylactic colectomy could be recommended to minimize the risk of CRC [31]. However, patients and clinicians have balked at this extreme solution as UC has a relatively low absolute risk of CRC over a lifetime, and a permanent ileostomy or restorative proctocolectomy significantly affects their quality of life, so they have sought ways to minimize CRC mortality and unnecessary colectomies by reducing both [31]. In patients with longstanding, extensive colitis, periodic colonoscopies combined with multiple biopsies of the colitic mucosa proved to be an effective way to detect dysplasia [32]. However, this surveillance has some limitations [31]. The lack of reasonable level of agreement between pathologists, patients under surveillance lost to follow-up, gastroenterologists with poor knowledge of dysplasia, endoscopists who practice insufficient biopsy techniques, and the absence of surgical recommendations upon obtaining a positive biopsy result are limitations that demonstrate the weaknesses of conventional surveillance for dysplasia as a cancer prevention method [31]. A retrospective cohort was conducted by Ullman that was published in 2003. The study analyzed Mount Sinai Gastrointestinal Pathology (New York City) databases of 46 UC patients diagnosed with low-grade flat dysplasia at surveillance colonoscopy between 1994 and 2001 [33]. It was carried out by compiling neoplastic progression rates and the frequency of advanced neoplasia [33]. It was found that unexpected advanced neoplasia occurred in 23.5% of patients, and the neoplastic progression rate was 53% at five years [33]. Cancers also developed despite frequent follow-up examinations (two of which had advanced stages) [33]. The authors concluded that the finding of low-grade flat dysplasia in patients with UC was a strong predictor of progression from dysplasia to advanced neoplasia and that early colectomy should be recommended for patients with UC with low-grade flat dysplasia [33]. Although accepting surgery is not easy for patients with asymptomatic dysplasia, it may be interesting to analyze the chance of dysplasia becoming a CRC in those who refused surgery. Murphy et al. published a retrospective review in 2016, carried out between 1993 and 2012, in the United States on a sample of 175 patients undergoing surgery for UC [34]. Progression from indeterminate dysplasia to low-grade dysplasia was observed in 20.6% of patients [34]. In 10.3% of patients, initial indeterminate dysplasia transformed into high-grade dysplasia [34]. Furthermore, 9.7% of patients with low-grade dysplasia progressed to high-grade dysplasia [34]. Moreover, the authors found no progression to CRC in patients with high-grade dysplasia [34]. The study also concluded that the incidence of CRC is low in patients with dysplasia if they postponed surgery for up to five years [34]. The results found in this study may help asymptomatic patients with UC-associated dysplasia who must choose between intensive surveillance or surgical intervention [34]. Another retrospective cohort conducted by Lim et al. between 1978 and 1990, published in 2003, can reinforce the results of the previous study [35]. This study was composed of 160 patients with longstanding, extensive UC recruited for annual colonoscopic monitoring [35]. The results of this cohort were interpreted 10 years after the end of the original study [35]. It was found after 10 years that the risk of developing high-grade dysplasia or CRC in patients with low-grade dysplasia was 10% and 4.0% in patients without baseline dysplasia [35]. Additionally, according to Kaplan-Meier analysis, there was no statistically significant difference in terms of mortality or postcolectomy complications between the two groups [35]. The study also concluded that the diagnosis of low-grade dysplasia does not justify a prophylactic colectomy because, after a 10-year evolutionary period, only 10% of patients with low-grade dysplasia will develop high-grade dysplasia or CRC [35]. Moreover, low-grade-dysplasia cases should not be ruled out for conservative management [35].

UC patients are recommended to use medical management as their first-line treatment; however, surgical treatment may be necessary for up to 25% to 30% of those affected by UC [36,37]. The primary treatment strategy for UC remains medical treatment, even though surgery offers a curative solution [38]. Surgery is indicated for various reasons in the treatment of UC [39]. These indications include fulminant colitis, perforation, toxic megacolon, lack of tolerance or inadequate response to medical treatment, presence of dysplasia or malignancy, growth retardation in children due to the disease, and improvement of extra-intestinal manifestations of the disease (e.g., uveitis) [39]. Table 2 summarizes the indications of surgery in UC.

**Table 2:** Indication of surgery in UC. UC, ulcerative colitis

Elective surgery in UC	Emergent surgery in UC
Dysplasia	Perforation
Growth retardation	Fulminant/toxic colitis
Medication intolerance	Toxic megacolon
Medically refractory disease	Medically refractory disease
Extraintestinal manifestations	Severe hemorrhage

In general, strict indications are necessary for emergent surgery due to the significantly higher risk of complications associated with such procedures [40]. Ikeuchi et al. conducted a study published in 2014 on the prognosis of elderly patients with UC following emergency surgery [40]. It turned out that 26.7% of emergency-operated patients died within 30 postoperative days, while just 0.88% of elective surgery patients died within 30 postoperative days [40]. The study also concluded that the prognosis is unfavorable following emergency surgery in elderly patients with UC [40]. Table 3 summarizes the studies regarding elective colectomy in UC surgical procedures for CRC.

**Table 3:** Summary of included studies regarding elective colectomy in UC surgical procedures for CRC. UC, ulcerative colitis; CRC, colorectal cancer; L-STC, laparoscopic subtotal colectomy; L-TPC, laparoscopic total proctocolectomy

References	Year of publication	Design	Number of participants	Population	Conclusion
Abd El Aziz et al. [48]	2021	-	3,387	Patients with UC who underwent elective total proctocolectomy or subtotal colectomy	Despite minimally invasive surgery, UC surgery is accompanied by a high rate of prolonged length of stay and blood transfusion. The operative approach has a greater impact on short-term outcomes and length of stay compared to the extent of resection. Compared to L-STC, L-TPC has higher rates of prolonged length of stay.
Leeds et al. [41]	2017	-	69,936	Patients with UC who had a non-elective total colectomy	Postoperative complications, hospital stay length, and hospital costs are all increased with delayed surgery for acute UC.
Khan et al. [46]	2017	A retrospective cohort study	59	Patients with UC who underwent partial resection or total proctocolectomy for CRC	Partial resection for CRC in UC may be a viable solution in a selected category of patients (particularly in elderly patients).
Lindberg et al. [47]	2006	-	210	Patients with UC who were followed by regular colonoscopies and biopsies	Limited resection of the colon and/or rectum increases the time with a better bowel function without increasing the risk of death from CRC and, therefore, may be considered an alternative to total proctocolectomy in patients with UC.

It has been shown that the postoperative outcomes of acute UC are improved following early surgical intervention due to the occurrence of fewer postoperative complications (the operation being defined as early, if it is performed within 24 hours of admission and delayed if carried out after 24 hours from admission) [41]. Leeds et al. conducted a study published in 2017 to see if the timing of emergency surgery in acute UC had a role in the outcome of surgery [41]. The study was conducted over 12 years (between 2002 and 2014) in a population sample of 69,936 patients with UC with a non-elective total colectomy identified in the National Inpatient Sample [41]. The results of patients who had early and delayed surgery were compared [41]. It was found that patients with delayed surgery had a greater risk of complications, higher hospital costs, and longer lengths of stay [41]. The study concluded that postoperative complications, hospital stay length, and hospital costs are all increased with delayed surgery for acute UC [41]. The findings of the above study can be compared along the lines of another study that was conducted by Patel et al. in 2013, which rested upon the conclusion that even if the medicinal therapeutic management of UC has very good results, UC is still associated with high morbidity and mortality and emergency surgery remains topical for the management of complications of UC [42]. The above studies can be contrasted with another study conducted by Tiu et al. in 2021, which found no significant difference in wound infection, sepsis, reoperation, or readmission between prompt and delayed surgery [43]. In conclusion, the study contradicts the two previous studies by referring that short-term outcomes are unaffected by delaying surgery for medical rescue therapy and that healing surgery may be proposed later when the general condition improves [43]. Ultimately, a retrospective matched cohort study conducted by Bewtra et al. in 2015 concluded that elective colectomy was associated with improved survival in patients aged 50 or older with advanced UC [44].

Finally, the treatment of UC by conservative therapy is often more effective. However, surgical treatment should not be viewed solely as a means of treating the negative disease progressions, but rather as a viable alternative. In certain cases, prolonged conservative treatment may be appropriate [45]. Furthermore, to optimize the timing and future of patients with UC, close collaboration between the different disciplines in pre- and postoperative management is essential [45].

Surgical approaches for the management of CRC and associated postoperative complications

Analyzing the previous articles, we have seen that colectomy is a good option for patients with UC who present with dysplasia [31]. For patients who choose the surgical option, different surgical procedures can be performed, all of which have certain advantages and disadvantages.

Khan et al. performed a retrospective cohort study, in 2017, to compare the results of segmental resection as an alternative to total proctocolectomy (TPC) in elderly patients with UC and malignancy [46]. The study included 59 patients with UC who underwent partial resection or TPC for CRC [46]. The study's endpoint was the development of metachronous cancer in patients who had undergone partial resection [46]. Of the 59 patients in the study, 24 had a partial resection and 35 had a TPC [46]. The authors found that no patient developed metachronous colon cancer of the retained colon during the median follow-up period of seven years after partial colectomy [46]. The study also concluded that performing partial resection for CRC in UC may be a viable solution in a selected category of patients (particularly elderly patients) [46]. Lindberg et al. conducted a study that was published in 2006 with a focus on patients with neoplastic changes; the study aimed to examine the outcome of patients undergoing limited resection of the colon and rectum instead of TPC [47]. The study was conducted over 29 years in a sample population of 210 patients with UC, followed by regular colonoscopies and biopsies [47]. Fifty-one patients with UC who presented with severe treatment-resistant disease, high-grade dysplasia, CRC, or repeated signs of low-grade dysplasia were operated on [47]. In 22 patients, resection of the colon or rectum was performed [47]. It was found that after performing a partial resection instead of a TPC, 21 patients gained an average of 9.4 years with better bowel function [47]. Additionally, no patient who received a partial resection died from metachronous cancer or CRC in their remaining colon or rectum [47]. The study also concluded that in patients with UC requiring surgery, performing a limited resection of the colon and rectum increased the time with a better bowel function without increasing the risk of death from CRC and, therefore, may be considered an alternative to TPC (Table 3) [47].

There are various surgical approaches available for the treatment of UC, and the ongoing debate surrounding them depends on the patient's profile and the associated complication rates. The choice of surgical approach plays a crucial role in determining the outcome and potential complications. Abd El Aziz et al. conducted a study that was published in 2021, with the aim of better understanding the impact on the short-term outcome of the surgical approach (laparoscopic or open surgery) and the extent of resection in patients with UC undergoing TPC and subtotal colectomy (STC) [48]. A sample of 3,387 patients with UC who underwent elective TPC or STC was selected from the American College of Surgeons-National Surgical Quality Improvement Program (ACS-NSQIP) database during 2011-2018 [48]. Patients were divided into four cohorts: open TPC (O-TPC), laparoscopic TPC (L-TPC), open STC (O-STC), and laparoscopic STC (L-STC) [48]. It was found that patients with O-TPC and those with O-STC had a similar risk of complication [48]. Additionally, patients who have had open surgery were known to have a greater risk of complications and longer hospitalization [48]. The authors also noted that patients with L-TPC had a more extended stay than those with L-STC, but this stay was shorter than those with O-STC [48]. The study concluded that despite minimally invasive surgery (such as laparoscopy), UC surgery was accompanied by a high rate: 27% more prolonged stay and 9% needed a blood transfusion [48]. The operative approach has a more significant impact on short-term outcomes and length of stay than the extent of resection [48]. Moreover, compared to L-STC, L-TPC has higher rates of prolonged length of stay (Table 3) [48].

It may be interesting to know if laparoscopic surgery can reduce the risk of postoperative complications. Tajti et al. conducted a study published in 2015 to analyze the long-term results of laparoscopic surgery in patients with UC [49]. The study was conducted for nine years (between 2005 and 2014) in Hungary in a population sample of 56 patients with UC who underwent surgical treatment [49]. In 33 patients, the laparoscopic method was used, and 23 patients were operated on by open surgery [49]. It was found that the two groups did not differ in hospital and intensive care unit stays bowel function recovery, and need for transfusions during the postoperative period [49]. However, bowel obstruction, sepsis, and other complications were significantly lower in patients who underwent laparoscopy than in open surgery [49]. The study also concluded that we could achieve better cosmetic results and a higher quality of life with laparoscopy [49]. Complications were also less common over time than in open surgery [49]. The previous results were completed by a second retrospective multicenter study conducted by Cai et al. and published in 2022 over nine years (between 2008 and 2017) in China on a population sample of 446 patients with UC surgery [50]. Laparoscopic surgery was associated with fewer short-term complications [50]. The study also concluded that during the past decade, surgical UC patients in China experienced fewer short-term postoperative complications, possibly due to the promotion of minimally invasive techniques by Chinese surgeons [50]. Preoperative corticosteroid treatment increases reoperation and complications risk after proctocolectomy, whether elective or non-elective [51]. It may be interesting to see if laparoscopic surgery would improve the outcome of surgery in UC patients with chronic preoperative steroid use. Lo et al. published a study in 2021 aiming to show that the use of laparoscopic surgery in UC patients with chronic preoperative steroid use would reduce the risk of septic shock/sepsis [52]. The study was conducted over 14 years (between 2005 and 2019) on a population sample of 8,644 patients with UC who underwent TPC identified from the National Surgical Quality Improvement Program (NSQIP) database from the American College of Surgeons [52]. Steroid use was present in 67.1% of patients, and nonsteroid use was present in 32.9%. Compared to open surgery, laparoscopic total abdominal colectomy performed on UC patients using chronic steroids demonstrated a reduced septic shock/sepsis risk [52]. The study concluded that patients with UC treated with chronic steroids before surgery were less likely to have septic shock or sepsis after laparoscopic surgery, suggesting that laparoscopic surgery may be a promising option for patients with preoperative chronic steroid use [52]. Table 4 summarizes the studies regarding postoperative complications in UC.

**Table 4:** Summary of included studies regarding postoperative complications in UC. UC, ulcerative colitis; CRC, colorectal cancer

References	Year of publication	Design	Number of participants	Population	Conclusion
Cai et al. [50]	2022	A retrospective multicenter study	446	Patients with surgery for UC in China	Surgical UC patients in China experienced fewer short-term postoperative complications, possibly due to the promotion of minimally invasive techniques by Chinese surgeons.
Lo et al. [52]	2021	-	8,644	Patients with UC who underwent total abdominal colectomy	Patients with UC treated with chronic steroids before surgery were less likely to have septic shock or sepsis after laparoscopic surgery, suggesting that laparoscopic surgery may be a promising option for patients with preoperative chronic steroid use.
Kohyama et al. [55]	2021	A retrospective multicenter study	5,284	Patients with UC after colectomy in Japan	Severe UC-related enteritis was a rare but serious complication after colectomy and could endanger the patient's vital prognosis.
Feuerstein et al. [56]	2018	-	258	Patients who had surgery for UC	There was no surgery-related mortality for UC at 30 days and very low surgery-related mortality at 90 days after surgery.
Ramsey et al. [53]	2017	-	397,847	Adult patients (>18 years old) with UC or Chron's disease who had had surgery for CRC	The outcome of CRC surgery would be negatively impacted if the patient had inflammatory bowel disease.
Wilson et al. [54]	2015	A matched cohort analysis	96,999	Patients undergoing colonic resection for diverticulitis, CRC, benign neoplasms, UC, and Crohn's disease	A very high risk of venous thromboembolism was present in hospitalized patients with UC undergoing colonic resection, and this risk always remained high in the period following discharge.
Tajti et al. [49]	2015	-	56	Patients with UC who underwent surgical treatment in Hungary	With laparoscopy, we could achieve better cosmetic results and a higher quality of life. Complications were also less common over time than with open surgery.

Additionally, the outcome of surgery for CRC will be impacted if the patient has IBD (including UC) [53]. In the study by Ramsey et al. published in 2017, which compared the results of surgery for CRC between patients with and without IBD, a sample of 397,847 adult patients (over the age of 18 years) with UC or Chron's disease who had had surgery for CRC were selected using National Inpatient Sample (2008-2012) and Nationwide Readmissions Database (NRD, 2013) [53]. It was found that patients with IBD who were operated on for CRC had a more extended hospital stay, were more likely to develop postoperative complications (such as postoperative infections and deep vein thrombosis), and required more frequent blood transfusions compared to patients without IBD [53]. The study also concluded that the outcome of CRC surgery would be negatively impacted if the patient had IBD (Table 4) [53].

Patients with UC undergoing colectomy are at high risk of developing venous thromboembolism [54]. During a follow-up period between 2005 and 2011, a matched cohort analysis performed by Wilson et al. in 2015 concluded that a very high risk of venous thromboembolism is present in hospitalized patients with UC undergoing colonic resection and that this risk always remains high in the period following discharge [54]. The findings of the above study can be compared along the lines of another study that was conducted by Kohyama et al. in Japan in 2021, a retrospective multicenter study from 2001 to 2014, revealed UC-related enteritis is a serious but rare complication after colectomy, which can endanger the patient's vital prognosis [55]. The studies above can be compared to a third study created by Feuerstein et al. in 2018, which affirmed that elective and non-elective surgery for UC is followed by rare mortality but that slight postoperative complications can appear [56]. The study was conducted over 12 years (between 2002 and 2014) on a population sample of 258 patients who had surgery for UC [56]. It was found that within 30 days of the operation, all patients were alive, and three patients died within 90 days of the operation [56]. The study concluded no surgery-related mortality for UC at 30 days and very low surgery-related mortality at 90 days after surgery (Table 4) [56].

Limitations

In this review, we analyzed studies that depict the current standing of surgery in UC in the context of increasingly effective medicamentous therapy. However, UC has various clinical presentations from one patient to another, with very mild to severe forms. Furthermore, this study does not address the very mild clinical presentation of the disease, which is nevertheless present in a large proportion of affected patients. Another major limitation of this review is that it only includes studies from PubMed; other databases were not accessed.

## Conclusions

As proven by the studies reviewed in this study, despite significant progress in the medicamentous treatment of UC in recent years, surgery still has a significant role for patients with UC. To summarize, the clinical significance of this review article is to establish a strong connection between UC and its surgical management in preventing the onset of complications such as perforation, life-threatening hemorrhage, toxic megacolon, pseudopolyps, strictures, and CRC (the most severe complication of UC). This study can operate as a tool for patients and physicians in making a therapeutic choice between annual colonoscopic surveillance and performing a preventive colectomy to prevent the onset of UC complications (a choice that is not easy to make). We mainly refer to challenges the physicians face while approaching the treatment of patients with UC, like educating patients about the severe complications that may occur in UC, the best choice between annual surveillance colonoscopy and preventive colectomy to avoid the occurrence of these complications, and whether surveillance colonoscopy is safe to diagnose dysplasia requiring surgery if knowing this procedure is dependent on the doctor. Furthermore, overcoming the obstacles in the management of UC is possible through a carefully structured treatment regimen tailored to the patient's specific factors. It is important not to overlook the option of surgery, which can prevent late complications of the disease and serve as a definitive treatment for UC. Finally, to emphasize the role of surgery in UC, further research studies are needed to identify cases of UC requiring surgical treatment.

## References

[1] Ordás I, Eckmann L, Talamini M, Baumgart DC, Sandborn WJ (2012). Ulcerative colitis. Lancet.

[2] Wei SC, Sollano J, Hui YT, Yu W, Santos Estrella PV, Llamado LJ, Koram N (2021). Epidemiology, burden of disease, and unmet needs in the treatment of ulcerative colitis in Asia. Expert Rev Gastroenterol Hepatol.

[3] Knyazev AV, Shkurko TV, Vardanyan AV, Romanov RI (2021). Surgical treatment of ulcerative colitis. History. Probl Sotsialnoi Gig Zdravookhranenniiai Istor Med.

[4] Clark-Snustad K, Butnariu M, Afzali A (2020). Women's health and ulcerative colitis. Gastroenterol Clin North Am.

[5] da Silva BC, Lyra AC, Rocha R, Santana GO (2014). Epidemiology, demographic characteristics and prognostic predictors of ulcerative colitis. World J Gastroenterol.

[6] Mendeloff AI, Monk M, Siegel CI, Lilienfeld A (1966). Some epidemiological features of ulcerative colitis and regional enteritis. a preliminary report. Gastroenterology.

[7] Rogers BH, Clark LM, Kirsner JB (1971). The epidemiologic and demographic characteristics of inflammatory bowel disease: an analysis of a computerized file of 1400 patients. J Chronic Dis.

[8] Molodecky NA, Soon IS, Rabi DM (2012). Increasing incidence and prevalence of the inflammatory bowel diseases with time, based on systematic review. Gastroenterology.

[9] Zhang YZ, Li YY (2014). Inflammatory bowel disease: pathogenesis. World J Gastroenterol.

[10] Danese S, Fiocchi C (2006). Etiopathogenesis of inflammatory bowel diseases. World J Gastroenterol.

[11] Kugathasan S, Fiocchi C (2007). Progress in basic inflammatory bowel disease research. Semin Pediatr Surg.

[12] Podolsky DK (2002). Inflammatory bowel disease. N Engl J Med.

[13] Nielsen OH, Jess T, Bjerrum JT, Seidelin JB (2014). Ulcerative colitis. Ugeskr Laeger.

[14] Chowdhury MS, Khan MR, Mahmuduzzaman M (2014). Disease extent and local complication of ulcerative colitis in bangladeshi population. Mymensingh Med J.

[15] Kaenkumchorn T, Wahbeh G (2020). Ulcerative colitis: making the diagnosis. Gastroenterol Clin North Am.

[16] Ekbom A, Adami HO, Helmick CG, Jonzon A, Zack MM (1990). Perinatal risk factors for inflammatory bowel disease: a case-control study. Am J Epidemiol.

[17] Langholz E, Munkholm P, Davidsen M, Binder V (1992). Colorectal cancer risk and mortality in patients with ulcerative colitis. Gastroenterology.

[18] Bernstein CN, Blanchard JF, Kliewer E, Wajda A (2001). Cancer risk in patients with inflammatory bowel disease: a population-based study. Cancer.

[19] Sjöqvist U (2004). Dysplasia in ulcerative colitis--clinical consequences?. Langenbecks Arch Surg.

[20] Nguyen GC, Frick KD, Dassopoulos T (2009). Medical decision analysis for the management of unifocal, flat, low-grade dysplasia in ulcerative colitis. Gastrointest Endosc.

[21] Kunovszki P, Milassin Á, Gimesi-Országh J (2020). Epidemiology, mortality and prevalence of colorectal cancer in ulcerative colitis patients between 2010-2016 in Hungary - a population-based study. PLoS One.

[22] Yashiro M (2014). Ulcerative colitis-associated colorectal cancer. World J Gastroenterol.

[23] Shah SC, Itzkowitz SH (2022). Colorectal cancer in inflammatory bowel disease: mechanisms and management. Gastroenterology.

[24] Itzkowitz SH, Yio X (2004). Inflammation and cancer IV. Colorectal cancer in inflammatory bowel disease: the role of inflammation. Am J Physiol Gastrointest Liver Physiol.

[25] Frick A, Khare V, Paul G (2018). Overt increase of oxidative stress and DNA damage in murine and human colitis and colitis-associated neoplasia. Mol Cancer Res.

[26] Olén O, Erichsen R, Sachs MC (2020). Colorectal cancer in ulcerative colitis: a scandinavian population-based cohort study. Lancet.

[27] Venkataraman S, Mohan V, Ramakrishna BS (2005). Risk of colorectal cancer in ulcerative colitis in India. J Gastroenterol Hepatol.

[28] Zhang Q, Sha S, Xu B, Liang S, Wu K (2015). Prevalence of colorectal cancer in patients with ulcerative colitis: a retrospective, monocenter study in China. J Cancer Res Ther.

[29] Issa IA, Noureddine M (2017). Colorectal cancer screening: an updated review of the available options. World J Gastroenterol.

[30] Carroll MR, Seaman HE, Halloran SP (2014). Tests and investigations for colorectal cancer screening. Clin Biochem.

[31] Ullman TA (2005). Preventing neoplastic progression in ulcerative colitis. J Clin Gastroenterol.

[32] Collins RH Jr, Feldman M, Fordtran JS (1987). Colon cancer, dysplasia, and surveillance in patients with ulcerative colitis. A critical review. N Engl J Med.

[33] Ullman T, Croog V, Harpaz N, Sachar D, Itzkowitz S (2003). Progression of flat low-grade dysplasia to advanced neoplasia in patients with ulcerative colitis. Gastroenterology.

[34] Murphy J, Kalkbrenner KA, Blas JV, Pemberton JH, Landmann RG, Young-Fadok TM, Etzioni DA (2016). What is the likelihood of colorectal cancer when surgery for ulcerative-colitis-associated dysplasia is deferred?. Colorectal Dis.

[35] Lim CH, Dixon MF, Vail A, Forman D, Lynch DA, Axon AT (2003). Ten year follow up of ulcerative colitis patients with and without low grade dysplasia. Gut.

[36] Metcalf AM (2007). Elective and emergent operative management of ulcerative colitis. Surg Clin North Am.

[37] Baker DM, Folan AM, Lee MJ, Jones GL, Brown SR, Lobo AJ (2021). A systematic review and meta-analysis of outcomes after elective surgery for ulcerative colitis. Colorectal Dis.

[38] Chen JH, Andrews JM, Kariyawasam V (2016). Review article: acute severe ulcerative colitis - evidence-based consensus statements. Aliment Pharmacol Ther.

[39] Albrechtsen D, Bergan A, Nygaard K, Gjone E, Flatmark A (1981). Urgent surgery for ulcerative colitis: early colectomy in 132 patients. World J Surg.

[40] Ikeuchi H, Uchino M, Matsuoka H (2014). Prognosis following emergency surgery for ulcerative colitis in elderly patients. Surg Today.

[41] Leeds IL, Truta B, Parian AM (2017). Early surgical intervention for acute ulcerative colitis is associated with improved postoperative outcomes. J Gastrointest Surg.

[42] Patel SS, Patel MS, Goldfarb M, Ortega A, Ault GT, Kaiser AM, Senagore AJ (2013). Elective versus emergency surgery for ulcerative colitis: a National Surgical Quality Improvement Program analysis. Am J Surg.

[43] Tiu SP, Hajirawala LN, Leonardi C, Davis KG, Orangio GR, Barton JS (2021). Delayed surgery does not increase risk in urgent colectomy for ulcerative colitis. Am Surg.

[44] Bewtra M, Newcomb CW, Wu Q (2015). Mortality associated with medical therapy versus elective colectomy in ulcerative colitis: a cohort study. Ann Intern Med.

[45] Kuehn F, Hodin RA (2018). Impact of modern drug therapy on surgery: ulcerative colitis. Visc Med.

[46] Khan N, Cole E, Shah Y, Paulson EC (2017). Segmental resection is a safe oncological alternative to total proctocolectomy in elderly patients with ulcerative colitis and malignancy. Colorectal Dis.

[47] Lindberg J, Stenling R, Palmqvist R, Rutegård J (2006). Surgery for neoplastic changes in ulcerative colitis--can limited resection be justified? Outcome for patients who underwent limited surgery. Colorectal Dis.

[48] Abd El Aziz MA, Perry WR, Grass F, Merchea A, Raffals LE, Mathis KL, Behm KT (2021). The extent of colorectal resection and short-term outcomes in patients with ulcerative colitis. Updates Surg.

[49] Tajti J Jr, Simonka Z, Paszt A (2015). Minimally invasive surgical treatment of ulcerative colitis--long-term results. Orv Hetil.

[50] Cai Z, He X, Gong J (2023). Laparoscopic surgery contributes to a decrease in short-term complications in surgical ulcerative colitis patients during 2008-2017: a multicenter retrospective study in China. Intest Res.

[51] Balachandran R, Tøttrup A (2015). Safety of proctocolectomy for ulcerative colitis under elective and non-elective circumstances: preoperative corticosteroid treatment worsens outcome. Dig Surg.

[52] Lo BD, Stem M, Zhang GQ (2021). The reduced risk of septic shock/sepsis with laparoscopic surgery among ulcerative colitis patients with preoperative chronic steroid use. Surgery.

[53] Ramsey M, Krishna SG, Stanich PP (2017). Inflammatory bowel disease adversely impacts colorectal cancer surgery short-term outcomes and health-care resource utilization. Clin Transl Gastroenterol.

[54] Wilson MZ, Connelly TM, Tinsley A, Hollenbeak CS, Koltun WA, Messaris E (2015). Ulcerative colitis is associated with an increased risk of venous thromboembolism in the postoperative period: the results of a matched cohort analysis. Ann Surg.

[55] Kohyama A, Watanabe K, Sugita A (2021). Ulcerative colitis-related severe enteritis: an infrequent but serious complication after colectomy. J Gastroenterol.

[56] Feuerstein JD, Curran T, Alosilla M, Cataldo T, Falchuk KR, Poylin V (2018). Mortality is rare following elective and non-elective surgery for ulcerative colitis, but mild postoperative complications are common. Dig Dis Sci.

